# A randomized, double-blind, placebo-controlled, repeated-dose pilot study of the safety, tolerability, and preliminary effects of a cannabidiol (CBD)- and cannabigerol (CBG)-based beverage powder to support recovery from delayed onset muscle soreness (DOMS)

**DOI:** 10.1080/15502783.2023.2280113

**Published:** 2023-11-10

**Authors:** Erica N. Peters, Helena Yardley, Amy Harrison, Graham M.L. Eglit, Jose Antonio, Cynthia Turcotte, Marcel O. Bonn-Miller

**Affiliations:** a Canopy Growth Corporation, Smiths Falls, Ontario, Canada; b Nova Southeastern University, Exercise and Sport Science, Davie, FL, USA; c Charlotte’s Web, Inc, Louisville, CO, USA

**Keywords:** Cannabidiol, cannabigerol, recovery, DOMS, delayed onset muscle soreness

## Abstract

**Background:**

Cannabinoid-containing products are marketed to athletes as promoting recovery, in spite of a lack of data on their safety and effects. This randomized, double-blind, placebo-controlled, repeated-dose pilot study tested the safety, tolerability, and preliminary effects on recovery of a formulation containing cannabidiol (CBD; 35 mg), cannabigerol (CBG; 50 mg), beta caryophyllene (BCP; 25 mg), branched-chain amino acids (BCAAs; 3.8 g), and magnesium citrate (420 mg).

**Methods:**

Exercise-trained individuals (*N* = 40) underwent an experimental induction of delayed onset muscle soreness (DOMS) and completed follow-up visits 24-, 48-, and 72-hours post-DOMS. Participants were randomized to active or placebo formulation, and consumed the formulation twice per day for 3.5 days.

**Results:**

There was one adverse event (AE) in the active group (diarrhea) and two AEs in placebo (dry mouth; eye rash/swollen eye). There was 100% self-reported compliance with formulation consumption across the two groups. For the primary outcome of interest, the estimate of effect for ratings of average soreness/discomfort 72 hours post-DOMS between active and placebo groups was −1.33 (85% confidence interval = -2.55, −0.10), suggesting moderate evidence of a treatment difference. The estimate of effect for the outcome of ratings of interference of soreness, discomfort, or stiffness on daily activities at work or home 48 hours post-DOMS was −1.82 (95% confidence interval = -3.64, −0.01), indicating a treatment difference of potential clinical importance. There was no significant effect between active and placebo groups on objective measures of recovery, sleep quality, or mood disturbance.

**Conclusions:**

The tested formulation reduced interference of DOMS on daily activities, demonstrating its improvement on a functional aspect of recovery.

## 1. Introduction

Interest in the use of cannabinoids among athletes is increasing. In a recent meta-analysis of > 46,000 athletes, ~23% had used cannabis and/or cannabinoids in the past year [1]. One of the most common reasons for use of cannabinoids among athletes is perceived ability to enhance recovery [2,3]. Products containing cannabinoids are available in the US and marketed to recreational and elite athletes (e.g. cbdMD™; ReCreate™) as promoting recovery, in spite of a small number of clinical trials on safety and effects of these products.

Cannabidiol (CBD) is a non-intoxicating cannabinoid that is commonly found in products promoting recovery. CBD acts through multiple mechanisms, and the extent of its molecular targets continues to be investigated. CBD is not a primary ligand of cannabinoid type 1 or type 2 receptors but may influence their signaling by modifying endocannabinoid tone [4]. CBD also interacts with many non-endocannabinoid signaling systems, such as the serotonin 1A (5-HT_1A_) receptor, the orphan G protein-coupled receptor 55, as well as the glycine, opioid, and peroxisome proliferator-activated receptors, various ion channels (e.g. the transient potential vanilloid receptor type 1 channel [TRPV1] and other transient potential vanilloid channels) and various enzymes (e.g. cyclooxygenase [COX]1 and COX2, cytochrome P450 enzymes) [5]. CBD has the potential to promote recovery via its reported anti-inflammatory and analgesic effects. More specifically, CBD may attenuate immune cell accumulation, stimulate production of anti-inflammatory cytokines, and inhibit production of pro-inflammatory cytokines and reactive oxygen species in preclinical models of acute inflammation, and has significant effects on analgesia in preclinical studies (see review of CBD and sports performance by McCartney et al. [6]). CBD has been evaluated in several human trials for its effects on recovery, with promising, although not completely consistent, results. Prior single-dose studies have demonstrated 16.67 mg CBD leads to lower ratings of soreness from 48 to 72 hours post-exercise; 60 mg CBD improves recovery of squat performance 72 hours, but not 24 or 48 hours, following resistance exercise and dampens the effect on muscle damage; and 300 mg CBD improves some, but not all, responses to aerobic exercise (increased respiratory gases, ratings of pleasure, and blood lactate concentrations) [7–9]. In the only repeated-dose study available, there was no effect of 150 mg CBD/day for 3 days on noninvasive measures of muscle damage following eccentric exercise [10].

Cannabigerol (CBG) is another cannabinoid that is increasingly found in products promoting recovery but, to our knowledge, has not been investigated in clinical studies. CBG could have anti-inflammatory properties because of its effects on adrenergic, 5-HT, TRPV, and cannabinoid receptors [11–13]. Older work supports that CBG has gamma aminobutyric acid (GABA) uptake inhibition greater than that of delta-9-tetrahydrocannabinol (THC) or CBD, suggesting muscle relaxant properties [14]. In one survey, a product containing 15 mg/mL CBG plus 5 mg/mL of CBD was associated with self-reported decreased fatigue, improved energy and thinking, improved attention levels, and improved anxiety, calmness, and sleep [15]. In another survey, a CBG-predominant product was associated with side effects of dry mouth, sleepiness, and increased appetite, reported by 16.5%, 15%, and 11.8% of survey respondents, respectively [16]. Although these surveys were not conducted among athletes, their preliminary findings point to initial safety of CBG and potential effects on aspects of recovery.

While scientific data on the anti-inflammatory and analgesic properties of CBD and CBG are accumulating, new products containing these hemp-derived cannabinoids are already being developed and sold. One strategy to optimize the effect on recovery of products containing CBD and CBG is to include other, commercially available, compounds that have recovery-enhancing properties. For example, beta caryophyllene (BCP) is a sesquiterpene (a type of terpene, or aromatic compound) found in cannabis and other plants that could have beneficial effects on recovery due to its anti-inflammatory effects, and its analgesic effects when combined with CBD [17–19], and branched-chain amino acids (BCAAs; valine, leucine, and isoleucine) and magnesium are non-cannabinoid ingredients that are commonly consumed by athletes for attenuation of muscle soreness [20,21]. Although both BCAAs and whole protein sources can attenuate muscle soreness, there is more literature to support the use of BCAAs in athletes and physically active individuals for recovery and muscle soreness, particularly with resistance training [20]. Furthermore, BCAAs require less volume compared to a whole protein such as whey, making them a pragmatic choice for inclusion in a multi-ingredient formulation. While the combination of these ingredients could theoretically promote recovery among athletes, a prudent approach before making such a formulation available is to examine its safety, tolerability, and preliminary effects. Examination of safety and tolerability outcomes is critical early in product development as there could be interactions among the multiple ingredients, which could lead to unexpected or unwanted side effects, or stronger or weaker effects than intended [22]. In the rapidly evolving landscape surrounding CBD, new products are entering the commercial market faster than scientific inquiry can keep pace. When considering the evaluation of numerous combinations of diverse ingredients at different dosages individually, it became apparent that such an approach would significantly delay the acquisition of essential empirical data regarding the safety and effects of final products. In light of this, we adopted a more efficient strategy: harnessing existing scientific literature to guide the selection of ingredients and their respective dosages that hold the greatest promise in alleviating DOMS, and then testing the finished formulation within an experimental, double-blind, randomized controlled trial. The current pilot study tested the safety, tolerability, and preliminary effects on recovery of a formulation that contained CBD, CBG, BCP, BCAAs, and magnesium, with the goal of leveraging results to inform a subsequent larger clinical study.

## 2. Materials and methods

### 2.1. Compliance with ethical standards

This study was conducted in accordance with consensus ethics principles, International Conference on Harmonization Good Clinical Practice guidelines, the Declaration of Helsinki, and local laws and regulations. The protocol was approved by the Advarra Institutional Review Board (Pro00055980; approved 4 November 2021). The study was registered on clinicaltrials.gov (NCT05026164; registered 30 August 2021). Written informed consent was obtained from each participant before any study-related procedures were performed.

### 2.2. Participants

Adults aged 18–65 years were eligible for the study if they were in good health as assessed by medical history; had a body mass index (BMI) 18–35 kg/m^2^; were “exercise-trained,” operationally defined as self-reported exercising at least 3 times per week for at least 30 minutes per session for the past year; and were willing to avoid exercise on the first day of the study and avoid weight lifting with upper extremities during the study. Females of childbearing potential were required to have a negative pregnancy test at screening. Females who were pregnant, lactating, breastfeeding, or planning a pregnancy were excluded, as were females of childbearing potential or males who were sexually active with females of childbearing potential who were unwilling or unable to use an acceptable method of contraception. To reduce variability in participants’ use of cannabinoids, participants could not have used cannabis, synthetic cannabinoid, cannabinoid analogues, hemp products, synthetic cannabinoid receptor agonists, or any CBD- or delta-9-tetrahydrocannabinol (THC)-containing products within 4 weeks of the first study visit or during the study; additionally, they could not have a positive urine drug screen prior to the first study visit. See Supplementary Table S1 for other exclusion criteria.

### 2.3. Study design

This randomized, double-blind, placebo-controlled, repeated-dose pilot study was conducted between December 2021 and February 2022 at one site in the United States. Individuals were recruited from panels of participants from past studies, local advertisements, and social media targeted advertisements. Individuals completed a telephone or online screening; those who met inclusion/exclusion criteria were enrolled into the study, scheduled for the first study visit, and randomized to active vs. placebo formulation condition in a 1:1 ratio.

At the first study visit, participants had their study eligibility confirmed and completed baseline assessments. To examine recovery, the first study visit involved an experimental induction of delayed onset muscle soreness (DOMS). DOMS is muscle soreness, pain, or discomfort that intensifies and peaks within one to 3 days following rigorous, unaccustomed exercise, and can also include a reduction in flexibility or mobility, decreased strength, increased fatigue, and decreased physical or athletic performance. In order for the study formulation to be in systemic circulation at the onset of the inflammatory process induced by the DOMS procedure, participants consumed the first dose of the study formulation 1 hour before undergoing the DOMS induction.

Following the DOMS induction and completion of the first study visit, participants were provided with 8 doses (7 scheduled doses + 1 extra dose in the event of lost/missing dose) of the study formulation to which they had been randomly assigned (i.e. active or placebo), with instructions on consumption of the study formulation at home. Participants completed 3 follow-up visits 24-, 48-, and 72 hours after the completion of the first study visit.

### 2.4. DOMS induction

The procedure to induce DOMS was the one repetition maximum (1RM) method of an eccentric contraction exercise from the non-dominant arm [23]. Each participant’s 1RM was established by determining the maximum amount of weight he/she lifted in a single repetition of a concentric exercise for the dominant elbow flexor muscles. Each participant performed 2–3 warm-up sets, and then made at most three attempts at a 1RM; the third 1RM was deemed the maximum 1RM for that participant. Approximately 5–10 minutes after the 1RM was determined, participants sat at a treatment table with a flexed shoulder joint at 45 degrees. Participants placed their elbow on the pad of the table, held a dumbbell weighing 60% of the 1RM, maintained it for one second at 90 degrees, and lowered it slowly for 3 seconds. After the elbow was completely extended, the experimenter manually put participants’ arm in a 90-degree flexion position. Using this method, participants completed 5 sets with 15 times as one set, and rested for 1 minute between sets. The induction was finished when participants completed 5 sets, or did not maintain extension of the elbow for 8 seconds.

### 2.5. Study formulation

The first step to determine compounds other than CBD and CBG to include in the study formulation was an environmental scan of compounds in commercially available recovery-based products. The next step was a comprehensive search of the published literature to collect scientific evidence on the recovery-promoting properties of compounds identified in the environmental scan. Results from published studies were documented, as were aspects of study methods that could impact the interpretation of results, including species, description of sample for studies of humans (e.g. exercise-trained or not), study design (e.g. clinical trial, experimental study of mechanism of action), sample size, dose of compound(s), description of exercise manipulation, reported safety signals, and outcome measures. Compounds were selected for inclusion in the study formulation based on strength of evidence regarding safety in humans and recovery-enhancing properties.

The active formulation was a powder in a 7.0 g single-serving packet that delivered 35 mg CBD, 50 mg CBG, 25 mg BCP, 3.8 g BCAAs, and 420 mg magnesium citrate. Because CBD and CBG are highly lipophilic molecules that do not dissolve well in water, the active formulation included HydroBond CBD™ and Hydrobond CBG™ water-soluble powders. These powders consisted of 20% of the given cannabinoid and 80% acacia gum and were manufactured into a nano-emulsion and spray dried to form a powder. The resulting powders were readily dispersible in liquid with no oily surfactants or bitter taste. Participants consumed 2 doses of study formulation per day; thus, the total daily consumption of active formulation was 70 mg CBD, 100 mg CBG, 50 mg BCP, 7.6 g BCAAs, and 840 mg magnesium citrate. Dosing was informed by federal agencies’ recommended daily limits, published data on tolerated doses in animals and effects on recovery in humans, and scans of the commercial market [24–30].

The placebo formulation was a powder in a 7.0 g single-serving packet that contained maltodextrin. To ensure similar flavor and color and thus maintain blinding, both active and placebo formulations contained passionfruit coconut natural flavor, Stevia, and beta carotene. Study personnel and participants were blinded to formulation assignment. Each participant only received one formulation in this parallel groups study design, thereby reducing the risk of direct comparison between active and placebo. Several steps were undertaken to ensure adequate blinding of study formulation. First, the study formulation was dispensed in single-serving sachets, with blinded labels. Second, the formulation contained water-soluble cannabinoids made from high purity distillates/isolates, not full spectrum extracts, thus limiting the distinctive smell and taste of cannabinoids. Third, flavoring, which included bitter blockers, was designed to mask the cannabinoids and provide a pleasant taste. Fourth, members of the research team and the team that formulated the active and placebo study formulations evaluated both formulations to ensure similarity, and no obvious visual features differed between formulations.

Participants were instructed to mix the powder with 16 oz. of water, and to consume orally. Participants were also instructed to consume the study formulation at 8 PM (±1 hour) the night of the first study visit, and then at 8 AM and 8 PM (±1 hour) every day until their final follow-up visit. The last dose occurred at 8 AM (±1 hour) immediately prior to the 72-hour post-DOMS study visit. To improve the bioavailability of cannabinoids, participants were recommended to consume the study formulation approximately 1 hour after completing a meal [31–35].

CBD and CBG were hemp-derived (derived from hemp containing no more than 0.3 percent THC on a dry weight basis) and thus were not controlled substances under U.S. federal law. The active and placebo powders were manufactured for the sponsor by a contract manufacturer according to current Good Manufacturing Practice (cGMP) 21 CFR part 111. Potency of cannabinoids (e.g. CBD, CBG) and within the final product was verified by both Botanacor (Denver, CO) and Eurofins (Madison, WI). Testing of BCP, magnesium citrate, and BCAAs was conducted at Eurofins (Madison, WI), and testing of contaminants (e.g. heavy metals, mycotoxins, pesticides, residual solvents, microbiology) was conducted by ACS Laboratory (Sun City, FL).

### 2.6. Safety and tolerability assessments

The safety assessment was adverse event (AE) and serious adverse event (SAE) monitoring at all visits. The tolerability assessment was self-reported compliance with study formulations consumption at all visits, and was calculated as the number of self-reported doses consumed/maximum of seven possible doses consumed.

### 2.7. Effects assessments

#### 2.7.1. Self-reported intensity and interference

Numeric rating scales (NRSs) were used to assess self-reported intensity of: (a) soreness or discomfort, and (b) stiffness, and self-reported interference of soreness, discomfort or stiffness on the ability to (a) perform daily activities at home or at work; and (b) participate in physical activities, pre-DOMS induction and at follow-up visits. Participants rated separate items of “average” and “worst” intensity over the last 24 hours. Each NRS was an 11-point scale (0–10) with anchors at, for example, 0 for “no soreness or discomfort” and 10 for “extreme soreness or discomfort.” These NRSs were developed for the current study, but similar scales have been used in previous studies to assess the effects of treatments on DOMS [36–39].

#### 2.7.2. Pressure threshold

Pressure threshold was measured with a 25-lb algometer pre-DOMS induction and at follow-up visits. Algometer pressure was applied perpendicular to the belly of the bicep at a slow and even speed. Participants were instructed to say “now” when the sensation of pressure changed to a sensation of pain. Pressure was applied 3 times 45 seconds apart for a total of 3 pressure threshold readings, and the average recording was used as the effect measure. The reproducibility, validity, and reliability of pressure threshold has been shown [40,41]. Pressure threshold has been used in previous studies to assess the effects of treatments on DOMS [42–45].

#### 2.7.3. Relaxed elbow angle

Hanging muscle tension was measured with a goniometer pre-DOMS induction and at follow-up visits. Participants stood comfortably with their arm hanging at their side. The goniometer was aligned with the acromion process and the midline of the ulna, with the joint of the goniometer centered over the lateral humeral epichondyle, and the resulting angle was recorded. Relaxed elbow angle has been used in previous studies to assess the effects of treatments on DOMS [36,46,47].

#### 2.7.4. Active range of motion

Participants’ exercised arm was placed on a table at a 90-degree angle. Participants were instructed to say “now” when they began to feel a painful pulling sensation in the bicep while extending their own arm outwards. The resulting angle was recorded with the goniometer, using the acromion process, midline of the ulna, and lateral humeral epichondyle as landmarks. This assessment was completed pre-DOMS induction and at follow-up visits. Active range of motion has been used in previous studies to assess the effects of treatments on DOMS [36,48].

#### 2.7.5. Passive range of motion

Participants’ exercised arm was placed on a table at a 90-degree angle. Participants were instructed to say “now” when they began to feel a painful pulling sensation in the bicep as the investigator extended the participant’s relaxed arm outwards. The resulting angle was recorded with the goniometer, using the acromion process, midline of the ulna, and lateral humeral epichondyle as landmarks. This assessment was completed pre-DOMS induction and at follow-up visits. Passive range of motion has been used in previous studies to assess the effects of treatments on DOMS [36,48].

#### 2.7.6. Sleep

A single-item NRS was used to assess sleep quality the previous night on an 11-point scale of 0–10 (0 = “worst sleep quality;” 10 = “best sleep quality”) pre-DOMS induction and at follow-up visits. The NRS was developed for the current study.

#### 2.7.7. Mood

The 65-item Profile of Mood States − 2 (POMS-2) was used to assess participants’ self-reported mood pre-DOMS induction and at follow-up visits [49,50]. Total mood disturbance score was calculated by adding scores on subscales of Tension, Depression, Anger, Fatigue and Confusion and then subtracting the Vigor subscale score.

### 2.8. Statistical analyses

Given its pilot nature, the study was not powered to detect a significant between-group effect. Instead, precision analysis was performed to determine the sample size required to achieve a suitable confidence interval width for treatment effect estimation. Average soreness or discomfort was designated as the primary effect of interest. Assuming a pooled standard deviation of 1.62 based on previous studies for average soreness/discomfort post-DOMS [51,52], a sample size of 32 was estimated to be required to achieve a 75% full confidence interval width of 1.35, an 85% full confidence interval width of 1.70, and a 95% full confidence interval width of 2.35. Accounting for 20% attrition, the required sample size was estimated to be 40 participants total.

Efficacy analyses were based on the modified intention-to-treat (mITT) population. The mITT was defined as all participants who were randomized to formulation group and received at least one dose of their assigned formulation and had baseline and at least one post-baseline efficacy evaluation. To estimate treatment effects, outcomes were analyzed using mixed models for repeated measures that assumed a first-order autoregressive covariance and homogeneous variance structure. Fixed effects consisted of baseline values of each outcome, treatment group, day, and the interaction of treatment group and day. Treatment effect estimates were calculated as contrasts between active and placebo groups adjusting for baseline outcome measures within each post-DOMS assessment.

To facilitate interpretation, standardized mean difference treatment effect measures were also calculated for all outcomes by dividing the raw treatment effect estimates derived from the analysis models described above by the average within-group standard deviation during the post-DOMS period [53]. We opted against using the baseline pre-DOMS pooled standard deviation of each outcome, as this measure of variability was not reflective of DOMS variability post-DOMS induction. Standardized mean differences were converted to Hedge’s g to adjust for small sample bias [54].

Following the recommendations of Lee et al. [55], both unstandardized and standardized effect size estimates were accompanied by 75%, 85%, and 95% confidence intervals. To determine the clinical potential of observed treatment effects, treatment effect point estimates and confidence intervals were interpreted in the context of the minimum clinically important difference (MCID) and the null effect of treatment (i.e. treatment effect of 0). For the primary effect of interest of average soreness/discomfort, the MCID was set at an unstandardized mean difference of −1.40 based on previous recommendation [56]. For all other outcomes, consensus MCIDs were not available, and an MCID of a standardized mean difference of −0.40 or 0.40 (depending on the direction of a favorable effect) was stipulated, assuming that a standardized effect size smaller than this would be unlikely to be clinically meaningful. A treatment effect whose confidence interval contained the null effect but not the MCID was deemed unlikely to be of clinical importance. A treatment effect whose confidence interval contained both the null effect and the MCID was equivocal. A treatment effect whose confidence interval contained the MCID but not the null effect was deemed of potential clinical importance. Finally, a treatment effect whose confidence interval exceeded the MCID and the null effect was deemed very likely to indicate a clinically important effect.

Safety analyses were conducted in the safety population, which was defined as all participants who were randomized to treatment and received study formulation. Safety analyses consisted of tabulation of incidence of AEs and SAEs reported by participants.

## 3. Results

### 3.1. Participant characteristics

Of the 40 participants randomized, all received study formulation and were included in the safety and mITT populations. Two participants (1 active, 1 placebo) did not complete the study for reasons not related to the study formulation or procedures; one participant dropped out due to a car accident and the other due to a scheduling conflict. Active and placebo groups were well-balanced on demographic and baseline characteristics (Table 1).

**Table 1.** Participant characteristics.

Characteristic	Placebo (*n* = 20)	Active (*n* = 20)	Overall (*n* = 40)
Age (years)	25.00 (6.46)	29.70 (10.14)	27.35 (8.73)
Sex [N (%) female]	11 (55)	8 (40)	19 (48)
Race [N (%)]			
Caucasian	15 (75)	16 (80)	31 (78)
Black or African American	4 (20)	3 (15)	7 (18)
Other	1 (5)	1 (5)	2 (5)
Ethnicity [N (%) Hispanic or Latino]	5 (25)	4 (20)	9 (23)
Body mass index	25.49 (4.61)	26.05 (3.95)	25.77 (4.24)
Percent fat mass	15.80 (8.00)	17.30 (7.43)	16.55 (7.66)
Percent lean body mass	58.38 (17.69)	60.94 (14.96)	59.66 (16.22)
Percent body fat	21.58 (8.57)	22.40 (8.47)	21.99 (8.42)
Total body water volume (L)	42.76 (12.96)	44.63 (10.97)	43.70 (11.89)
Type of exercise on the day preceding the first study visit [N (%)]			
Aerobic	1 (5.3)	5 (25)	6 (15)
Strength training	7 (37)	2 (10)	9 (23)
Aerobic and strength training	4 (21)	7 (35)	11 (28)
None	7 (37)	6 (30)	13 (33)
Duration of exercise on the day preceding the first study visit	41.90 (42.43)	50.75 (49.95)	46.33 (45.97)
Average soreness or discomfort	1.95 (1.93)	2.05 (2.11)	2.00 (2.00)
Average stiffness	1.70 (1.53)	1.80 (2.48)	1.75 (2.03)
Interference: Daily activities	1.10 (1.48)	0.75 (1.29)	0.92 (1.38)
Interference: Physical activity	1.75 (2.12)	1.20 (2.31)	1.48 (2.21)
Worst soreness or discomfort	2.35 (2.32)	3.25 (3.13)	2.80 (2.76)
Worst stiffness	2.15 (2.03)	2.35 (2.74)	2.25 (2.38)
Pressure threshold	59.02 (25.77)	57.77 (23.40)	58.40 (24.31)
Active range of motion	132.80 (26.29)	129.45 (21.83)	131.12 (23.91)
Passive range of motion	149.32 (9.05)	138.20 (27.79)	143.76 (21.17)
Muscle circumference	31.41 (5.38)	32.55 (4.07)	31.98 (4.74)
Relaxed elbow angle	15.12 (8.29)	16.25 (9.03)	15.69 (8.58)
Sleep quality	6.35 (2.13)	6.85 (1.87)	6.60 (2.00)
POMS-2 total mood disturbance score	19.85 (38.48)	4.15 (20.26)	12.00 (31.38)

*Note*. Unless otherwise noted, means (standard deviations) are presented. POMS-2: Profile of Mood States.

### 3.2. Safety and tolerability

In the active formulation group, one participant reported one AE of diarrhea that was rated as severe. The AE started on day 2 of study formulation consumption and resolved on day 3; the participant completed the study. In the placebo group, two participants reported one AE each, both of which were rated as mild. One participant reported dry mouth that started on day 2 of placebo formulation consumption and resolved on day 3; the participant completed the study. Another participant reported an eye rash/swollen eye that started on day 2 of placebo formulation consumption; this participant withdrew from the study on day 3, citing problems with transportation to study visits. There were no SAEs, no formulation-related AEs leading to treatment discontinuation, and no deaths in either group.

The rate of compliance with study formulation consumption was 100% among the 38 completed participants. The two participants who discontinued the study self-reported 100% compliance with study formulation until the point of study discontinuation.

### 3.3. Preliminary effects

**Figure 1.**
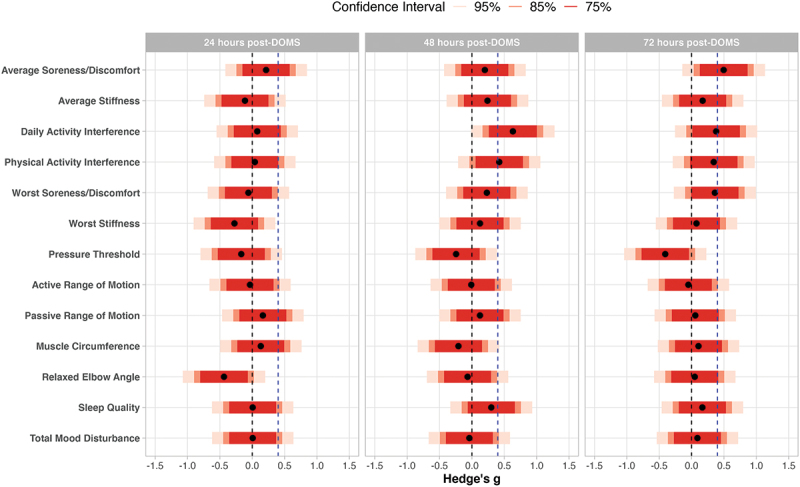
Standardized treatment effect estimates for preliminary effects. Effect estimates for average soreness/discomfort, average stiffness, daily activity interference, physical activity interference, worst soreness/discomfort, worst stiffness, muscle circumference, and relaxed elbow angle were reflected so that all positive effect estimates indicate active superior to placebo and all negative effect estimates indicate placebo superior to active. The dotted vertical blue line indicates minimally clinical important difference of 0.40; the dotted vertical black line indicates null effect.

Table 2 shows standardized and unstandardized treatment effect estimates, and Figure 1 displays standardized treatment effect estimates, for effects measures between active and placebo groups. The Supplementary Table provides descriptive means and standard deviations for effects measures between active and placebo groups.

**Table 2.** Unstandardized and standardized mean difference of active study formulation from placebo and standard errors.

	Unstandardized			Standardized		
	24 hrs post-DOMS	48 hrs post-DOMS	72 hrs post-DOMS	24 hrs post-DOMS	48 hrs post-DOMS	72 hrs post-DOMS
Average soreness or discomfort	−0.57 (.83)	−0.53 (.84)	−1.33 (.84)	−0.21(.31)	−0.20 (.31)	−0.50 (.32)
Average stiffness	0.31 (.86)	−0.66 (.87)	−0.47 (.87)	0.11 (.31)	−0.24 (.31)	−0.17 (.31)
Interference: Daily activities	−0.22 (.89)	−1.8 (.91)	−1.09 (.91)	−0.08 (.31)	−0.63 (.32)	−0.38 (.31)
Interference: Physical activity	−0.12 (.97)	−1.30 (.98)	−1.06 (.98)	−0.04 (.31)	−0.42 (.31)	−0.34 (.31)
Worst soreness or discomfort	0.18 (.97)	−0.71 (.98)	−1.10 (.98)	0.06 (.31)	−0.23 (.31)	−0.36 (.31)
Worst stiffness	0.81 (.92)	−0.37 (.93)	−0.22 (.93)	0.28 (.31)	−0.13 (.31)	−0.08 (.31)
Pressure threshold	3.34 (6.15)	4.81 (6.24)	8.01 (6.24)	0.17 (.31)	0.25 (.31)	0.41 (.31)
Active range of motion	0.48 (4.35)	0.14 (4.38)	0.69 (4.39)	0.03 (.31)	0.01 (.31)	0.05 (.31)
Passive range of motion	−1.78 (3.53)	−1.36 (3.57)	−0.63 (3.57)	−0.16 (.31)	−0.13 (.31)	−0.06 (.31)
Muscle circumference	−0.23 (.55)	0.36 (.55)	−0.18 (.55)	0.21 (.31)	−0.11 (.31)	−0.11 (.31)
Relaxed elbow angle	6.39 (4.58)	0.98 (4.63)	−0.72 (4.64)	0.44 (.31)	0.07 (.31)	−0.05 (.31)
Sleep quality	−0.01 (.57)	−0.62 (.59)	−0.34 (.58)	−0.01 (.31)	−0.30 (.31)	−0.17 (.31)
Total mood disturbance score	−0.23 (3.93)	−1.24 (4.01)	−2.84 (3.99)	−0.01 (.31)	0.04 (.31)	−0.09 (.31)

*Note*. Mean differences from placebo were not reflected for any outcome. DOMS: delayed onset muscle soreness.

**Figure 2.**
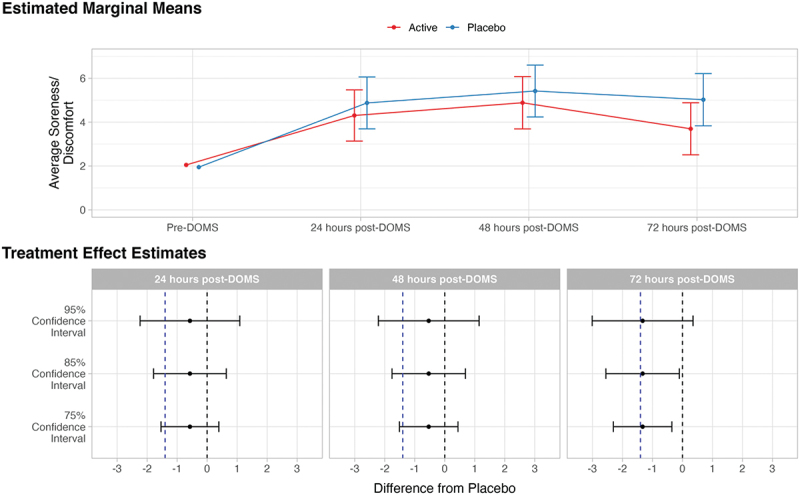
Estimated marginal means and treatment effect estimates for the effect of average soreness or discomfort. In the estimated marginal means panel, bars represent 95% confidence intervals. In the treatment effect estimates panel, the dotted vertical blue line indicates minimally clinical important difference of 1.40 (lv et al., 2020; Tashjian et al., 2009); the dotted vertical black line indicates null effect.

On the primary outcome of interest, average soreness/discomfort, treatment effect point estimates were in the favorable direction (lower average soreness/discomfort for active formulation vs. placebo) but were nonetheless below the MCID at 24- (estimated mean difference = −0.57), 48- (estimated mean difference = −0.53) and 72-hours post-DOMS (estimated mean difference = −1.33), although the 72-hour estimate was very close to the MCID of −1.40. At 24- and 48-hours post-DOMS, all confidence intervals (75%, 85%, and 95%) contained both the MCID and the null value, indicating equivocal results at these time points. At the 72-hour time point, the 95% confidence interval (−3.01, 0.35) crossed the MCID and 0, while the 75% (−2.30, −0.35) and 85% (−2.55, −0.10) confidence intervals crossed the MCID but not 0. This suggests moderate evidence in favor of a treatment difference 72 hours post-DOMS that is of potential clinical importance (Figure 2).

The treatment effect estimate for ratings of interference of soreness, discomfort, or stiffness on daily activities at work or home was above the MCID at 48 hours post-DOMS (g = −.63), and the 85% confidence interval (−1.10, −0.17) did not cross 0; this suggests a treatment difference of potential clinical importance. The treatment effect estimate for ratings of interference of soreness, discomfort, or stiffness on physical activities exceeded the MCID at 48 hours post-DOMS (g = −0.42) and the 75% confidence interval did not cross 0, again suggesting a potentially clinically important effect. For both outcomes, standardized treatment effects were slightly smaller at 72-hours post-DOMS, but close to the stipulated MCID (g = −0.34 to −0.38).

All treatment effect estimates for passive range of motion, muscle circumference, relaxed elbow angle, sleep quality, and total mood disturbance score were below the MCID at all post-DOMS timepoints and confidence intervals contained both the null effect and the MCID, indicating equivocal findings. Treatment effect estimates for pressure threshold and active range of motion were below the MCID (g = 0.03 to 0.41) at all time points and most confidence intervals failed to contain the MCID, suggesting a low likelihood of clinically important effects.

## 4. Discussion

The primary aim of this pilot study was to examine the safety and tolerability of a CBD- and CBG-based formulation that was developed to enhance recovery among exercise-trained individuals who self-reported exercising at least 3 times per week for at least 30 minutes per session for the past year. There were no safety concerns and no unexpected interactions among the multiple ingredients in the formulation. Self-reported compliance with formulation consumption over 3.5 days of dosing at home was excellent. Thus, the first test of this formulation in a small sample of exercise-trained individuals indicated a good safety and tolerability profile.

The formulation showed a signal for modest effects on decreased self-reported average soreness/discomfort 72 hours post-DOMS, complementing previous supportive findings of the effect of CBD on recovery [7–9]. While the formulation did not exert meaningful effects on objective measures of recovery, it reduced interference of self-reported soreness/discomfort on participants’ ability to perform daily activities at work or home, and to perform physical activity, at 48 hours post-DOMS. Thus, functional indicators of well-being could be enhanced by this new cannabinoid-based formulation. Improvement in functional domains may arguably be more indicative of the value of the formulation to consumers than reduced discomfort intensity may be, as reduced discomfort has less impact on an individual if functioning is not also improved [57]. Because the analytical approach was intended to inform the design of future larger trials, these observations on effects are preliminary and should be interpreted with caution [55].

CBG is an understudied cannabinoid that is increasingly found in products marketed to athletes as promoting recovery for its potential anti-inflammatory properties [11–13]. This is the first study of the effects of CBG on exercise-related outcomes and, to our knowledge, also the first clinical trial of the effects of CBG in humans. The experimental results reported here suggest good safety and tolerability, and a potential role in helping reduce muscle soreness/discomfort and reduce interference of muscle soreness/discomfort on performance of daily activities, when dosed at 50 mg twice per day and with the other ingredients found in the formulation. Future trials are needed to evaluate the safety and effects of other doses and other dosing regimens of CBG, and to identify the mechanisms by which CBG could help promote recovery.

Although a unique aspect of this pilot study was the examination of CBG in humans, CBG was only one ingredient in the evaluated formulation. Perhaps the most significant limitation of this study was that the formulation contained multiple ingredients, making it impossible to isolate which ingredient(s) drove preliminary effects on recovery. Another limitation is that formulation dosing prior to the DOMS induction may not reflect how individuals would consume a product for recovery-related purposes in the real world. This study recruited exercise-trained participants who self-reported exercising at least 3 times per week for at least 30 minutes per session for the past year, and findings may not generalize to non-exercise-trained individuals or to those with a different training volume. The study included a large number of outcomes across multiple time points. No adjustment to confidence interval widths was implemented because this was a pilot study; false positive interpretations of effect sizes as indicative of potential clinical meaningfulness are possible, especially for outcomes for which a consensus MCID is not established. Future evaluations in larger samples would benefit from assessment of whether the blinding of the study formulation was adequate (e.g. ask participants to guess which formulation they consumed and why they guessed that formulation), objective assessment of compliance with formulation consumption (e.g. cannabinoid concentrations in plasma and urine), and assessment of compliance with instructions on consumption of the formulation (e.g. whether the powder was mixed with water as instructed or with a different liquid; whether the participant consumed the formulation after a meal as instructed or on an empty stomach).

The findings of this pilot study have implications for design of future DOMS trials. The observed standard deviation on post-DOMS average/soreness was larger than anticipated based on previous literature. This may be a result of different DOMS induction methods in the current vs. some previous studies (upper body eccentric contraction vs. lower body eccentric contraction; running; cycling). Future trial design should select a standard deviation estimate for power/precision analysis based on prior studies using similar DOMS induction approaches. As complete symptom resolution was not achieved by the 72-hour assessment for most outcomes, future trials may extend the duration of follow-up to capture resolution of more persistent symptoms or evaluate possible cumulative effects over time. Future trials may adopt a dismantling design to compare the formulation tested here to its constituent ingredients and help determine which component(s) drive effects on recovery. Although effects on sleep and mood were not observed, a different pattern might emerge among exercise-trained individuals who report baseline disturbance in sleep or mood.

Results from this pilot study revealed a good safety and tolerability profile associated with a beverage powder containing CBD, CBG, BCP, BCAAs, and magnesium citrate. Preliminary results on effects suggested that repeated-dosing of this cannabinoid-based formulation over 3.5 days improved functional aspects of recovery, and could decrease self-reported soreness or discomfort, associated with DOMS.

## Supplementary Material

Supplemental Material
