# Supplementary material for: A randomized, double-blind, placebo-controlled, repeated-dose pilot study of the safety, tolerability, and preliminary effects of a cannabidiol (CBD)- and cannabigerol (CBG)-based beverage powder to support recovery from delayed onset muscle soreness (DOMS)
Source: J Int Soc Sports Nutr. 2023 Nov 10;20(1):2280113. doi: 10.1080/15502783.2023.2280113 (PMC10653658; doi:10.1080/15502783.2023.2280113)
Supplement: Supplemental Material [file RSSN_A_2280113_SM9281.docx]

Supplementary Information.

Exclusion criteria included females who were pregnant, lactating, breastfeeding or planning a pregnancy; females of childbearing potential, or males who were sexually active with females of childbearing potential, who were unwilling or unable to use an acceptable method of contraception; having a history of epilepsy, hepatitis, clinically significant hepatic or renal impairment, or human immunodeficiency virus; changes in the use of prescription or over-the-counter drugs, herbal supplements, or vitamins (except hormonal contraception) for the month prior to the first study visit, use of any analgesic drugs within 24 hours prior to the first study visit; having any clinically significant condition or abnormal finding at screening that would, in the opinion of the Investigator, preclude study participation or interfere with the evaluation of the study product; having a history or current diagnosis of a significant psychiatric disorder that would, in the opinion of the Investigator, affect the participant’s ability to comply with the study requirements; endorsement of current suicidal intent; a history of a known significant allergic condition, significant drug-related hypersensitivity, or allergic reaction to any compound or chemical class related to cannabis, including phytocannabinoids and cannabinoid analogues, or excipients utilized within the study product; having musculoskeletal issues that might impede performing maximal elbow flexion exercises; having taken a medication with likely CBD-interactions, including warfarin, clobazam, valproic acid, phenobarbital, mTOR inhibitors, oral tacrolimus, and St. John’s Wort within 30 days of the first study visit or during the study; having taken grapefruit products and/or Seville oranges within the 7 days prior to dosing with study product; use of cannabis, synthetic cannabinoid, cannabinoid analogues, hemp products, synthetic cannabinoid receptor agonists, or any CBD- or delta-9-tetrahydrocannabinol (THC)-containing products within 4 weeks of the first study visit or during the study; having participated in any investigational product or device study within 30 days prior to the first study visit, or was scheduled to participate in another investigational product or device study during the course of this study; and a positive urine drug screen prior to the first study visit.

Supplementary Table. Means and Standard Deviations for Preliminary Effects of Active vs. Placebo Study Product.

| Effect | Placebo | | | | Active | | | |
| --- | --- | --- | --- | --- | --- | --- | --- | --- |
|  | Pre-DOMS | 24 hrs post-DOMS | 48 hrs post-DOMS | 72 hrs post-DOMS | Pre-DOMS | 24 hrs post-DOMS | 48 hrs post-DOMS | 72 hrs post-DOMS |
| Average soreness/discomfort in the past 24 hours | 1.95 (1.93) | 4.84 (2.01) | 5.32 (2.91) | 4.95 (3.19) | 2.05 (2.11) | 4.40 (2.35) | 4.72 (2.40) | 3.53 (2.72) |
| Worst soreness/discomfort in the past 24 hours | 2.35 (2.32) | 5.32 (2.54) | 6.32 (3.00) | 5.53 (3.32) | 3.25 (3.13) | 5.60 (2.72) | 5.44 (2.77) | 4.32 (3.28) |
| Average stiffness in the past 24 hours | 1.70 (1.53) | 4.26 (2.68) | 5.32 (2.98) | 4.16 (3.00) | 1.80 (2.48) | 4.70 (2.47) | 4.50 (2.26) | 3.63 (2.91) |
| Worst stiffness in the past 24 hours | 2.15 (2.03) | 5.05 (2.61) | 6.05 (3.08) | 4.63 (3.11) | 2.35 (2.74) | 5.85 (2.70) | 5.50 (2.64) | 4.32 (3.37) |
| Interference in daily activities at work or home | 1.10 (1.48) | 3.37 (2.61) | 4.89 (3.02) | 3.74 (3.00) | 0.75 (1.29) | 3.30 (2.47) | 2.89 (2.47) | 2.63 (2.87) |
| Interference in physical activities | 1.75 (2.12) | 3.74 (3.11) | 4.74 (3.19) | 3.89 (3.18) | 1.20 (2.31) | 3.80 (2.57) | 3.28 (2.61) | 2.84 (3.06) |
| Pressure threshold | 59.02 (25.77) | 46.35 (18.88) | 44.27 (23.20) | 52.50 (22.49) | 57.77 (23.40) | 50.75 (18.27) | 47.89 (15.86) | 58.25 (25.45) |
| Active range of motion | 132.80 (26.29) | 130.53 (11.69) | 129.16 (16.17) | 131.05 (15.82) | 129.45 (21.83) | 131.05 (12.13) | 128.22 (14.04) | 131.16 (14.42) |
| Passive range of motion | 149.32 (9.05) | 145.42 (10.34) | 142.79 (12.91) | 143.58 (11.57) | 138.20 (27.79) | 142.95 (8.68) | 140.39 (12.34) | 142.11 (11.05) |
| Muscle circumference | 31.41 (5.38) | 31.45 (5.43) | 32.21 (5.35) | 32.54 (5.61) | 32.55 (4.07) | 32.77 (4.21) | 33.13 (4.49) | 33.28 (4.39) |
| Relaxed elbow angle | 15.12 (8.29) | 21.16 (13.99) | 26.89 (17.00) | 22.68 (16.35) | 16.25 (9.03) | 27.40 (15.15) | 27.28 (13.68) | 22.11 (11.30) |
| Sleep quality | 6.35 (2.13) | 6.05 (2.25) | 6.42 (1.68) | 7.05 (2.04) | 6.85 (1.87) | 6.35 (2.30) | 6.06 (2.10) | 6.89 (2.00) |
| POMS - 2 total mood disturbance score | 19.85 (38.48) | 15.05 (28.64) | 8.05 (20.89) | 12.16 (26.25) | 4.15 (20.26) | 4.35 (16.80) | 0.45 (10.10) | 4.28 (11.93) |

*Note*. DOMS: delayed onset muscle soreness; POMS - 2 : Profile of Mood States – 2.
